# Differential Expression of Hard Tissue Proteins in Hypomineralized Second Primary Molars in Comparison to Normal Teeth

**DOI:** 10.1002/cre2.70079

**Published:** 2025-02-03

**Authors:** Sharon Jessica, Ramya Sekar, Snehashish Ghosh, Safal Dhungel, Kavitha B., Mahesh Ramakrishnan, Shazia Fathima Jh, Monisha Prasad, Jaiganesh I., Sindhu Subramani

**Affiliations:** ^1^ Department of Oral and Maxillofacial Pathology and Oral Microbiology Meenakshi Ammal Dental College and Hospital, Meenakshi Academy of Higher Education and Research (Deemed to be University) Chennai Tamil Nadu India; ^2^ Department of Biochemistry Centre of Molecular Medicine and Diagnostics (COMManD), Saveetha Dental College & Hospital, Saveetha Institute of Medical & Technical Sciences Chennai Tamil Nadu India; ^3^ Department of Oral Pathology College of Medical Sciences Bharatpur Nepal; ^4^ Department of Oral and Maxillofacial Surgery College of Medical Sciences Bharatpur Nepal; ^5^ Department of Pedodontics & Preventive Dentistry Saveetha Dental College & Hospital, Saveetha Institute of Medical & Technical Sciences Chennai Tamil Nadu India; ^6^ Department of Oral Pathology & Microbiology Ragas Dental College & Hospital Chennai Tamil Nadu India; ^7^ Centre for Global Health Research, Saveetha Medical College, Saveetha Institute of Medical and Technical Sciences Chennai Tamil Nadu India; ^8^ Department of Pediatric & Preventive Dentistry Meenakshi Ammal Dental College and Hospital, Meenakshi Academy of Higher Education and Research (Deemed to be University) Chennai Tamil Nadu India

**Keywords:** dental caries, hypomineralization, molars, primary teeth

## Abstract

**Objective:**

This study aims to identify the proteins in hypomineralized second primary molars (HSPMs) and correlate their function in Amelogenesis. HSPM is a qualitative defect of the enamel of the second primary molars with no clear etiology.

**Material and Methods:**

Total protein quantification was performed using the Bradford Protein Assay, followed by the electrophoretic separation of samples using 2D‐Gel electrophoresis to identify the proteins.

**Results:**

The results from the Bradford Protein Assay unveiled a five‐fold increase in the protein content in HSPM. Proteins such as Dentin sialo‐phosphoprotein (DSPP), Keratin, type I, Serum Albumin, Anti‐thrombin III, Alpha‐1‐Antitrypsin, Histone H3.2, Actin, Heat shock Protein, Vimentin, Desmoglein‐3, Glyceraldehyde‐3‐phosphate dehydrogenase, Inosine‐5'‐monophosphate dehydrogenase 2, Zinc Alpha 2 glycoprotein, Lysozyme C, Prothrombin, Vit‐D binding Protein, Apolipoprotein A‐1, Defensin 1, Immunoglobulin Gamma, Immunoglobulin Kappa, and Alpha‐Amylase were all upregulated (*p* < 0.05) in HSPM.

**Conclusion:**

This investigation conclusively demonstrates that HSPM‐affected teeth have higher protein content than healthy teeth. The study also supports the theory of proteolytic inhibition attributed to reduced protease activity and heightened protease inhibitor activity.

## 1 Introduction

Hypomineralized second primary molar (HSPM), previously known as deciduous molar hypomineralization (DMH), is a qualitative defect of the enamel of the primary molars with no clear etiology. As it frequently occurs in second molars, Elfrink et al. (2015) coined the term “Hypomineralized Second Primary Molar.” Most of the time, HSPM acts as a predecessor lesion for MIH. By definition, HSPM is the “hypomineralisation involving one to four primary second molars with cheesy opacities, PEB (posteruptive breakdown), atypical restorations/caries, and extractions because of HSPM” (Elfrink et al. 2015). The prevalence of HSPM is highly varied due to variations in examination and diagnosis. A highly varied range of 0%–41% in the prevalence of HSPM was reported, with 6.8% pooled prevalence in children (McCarra et al. 2022). This varied prevalence data could be due to different diagnostic indices such as developmental defects of enamel (DDE), modified developmental defects of enamel (mDDE), MIH/HSPM index, and a few self‐devised indices. The presence of HSPM was reported to lead to significantly higher odds for the development of MIH in the future. MIH affects one in six children worldwide. Epidemiological studies from different parts of the world show a wide variation in the prevalence of MIH, ranging between 2.8% and 40.2% (Ghanim et al. 2017). The mean global prevalence is 13%, and 4.8 million cases per year require treatment (Schwendicke et al. 2018). MIH in the permanent dentition of Indian children was estimated to be 8.9%, with no gender predominance (Kirthiga et al. 2015).

Enamel hypoplasia and hypomineralization are two distinct categories of developmental abnormalities prevalent in both primary and permanent dentitions. A qualitative defect known as hypomineralization of enamel results from aberrations in the early maturative or secretory stage of enamel production (Weerheijm 2004; Almuallem and Busuttil‐Naudi 2018). The clinical manifestations of HSPM include post‐enamel breakdown (PEB), delimited opacities that range from white to brown, and obvious border changes in enamel translucency (Ghanim et al. 2015). Because HSPM resembles defined opacities in primary and permanent teeth, it can be difficult to diagnose clinically. By combining the updated DDE index with the recommendations of the European Academy of Paediatric Dentistry (EAPD), new diagnostic standards have been put forth to address this. As the primary second molar and permanent first molar development periods overlap, HSPM may be a precursor lesion to molar incisor hypomineralization (MIH), an uncommon dental condition with significant clinical implications (Negre‐Barber et al. 2016). Investigation of HSPM is essential for understanding the complexities of tooth growth and mineralization processes as well as for determining the cause of this disorder.

Many proteins, proteases, and regulatory factors work in concert to orchestrate the development of teeth. Conditions like HSPM may result from disruptions in the expression or function of molecular factors, which can have a significant impact on tooth mineralization (Elfrink et al. 2015). A more thorough analysis of the proteins involved in enamel and dentin growth, as well as the regulatory mechanisms controlling their activities, is necessary to comprehend the molecular mechanisms responsible for HSPM.

Several electrophoretic studies have been conducted on the enamel of normal teeth and it has been identified that the enamel is predominantly made up of non‐collagenous proteins such as amelogenins. This type of heterogenous group of low‐molecular‐weight proteins contributes to about 90% and the rest, 10%, is made up of non‐amelogenins such as ameloblastin and enamelin (Nanci et al. 2018). Apart from these proteins, proteases such as enamelysin and serine proteases such as kallikrein 4 play an important role in the extracellular processing of the aforementioned proteins. Other ameloblast products such as odontogenic ameloblast‐associated protein (ODAM) and amelotin are also found on the surface that undergo bulk degradation during the maturation process (Moradian‐Oldak 2012; Gibson 2011). The interplay between these proteins, proteases, and other ameloblast products results in decreased degradation of proteins, causing hypomineralization. Therefore, in this research, we have studied the differential expression of proteins by electrophoresis in Hypomineralized teeth in comparison to normal teeth to understand the proteomics of Hypomineralized primary second molar. Additionally, we investigated potential crosstalk between these proteins and protease inhibitors and their role in regulating protease activity during tooth development.

## 2 Materials and Methods

### 2.1 Study Design

**Figure 1:**
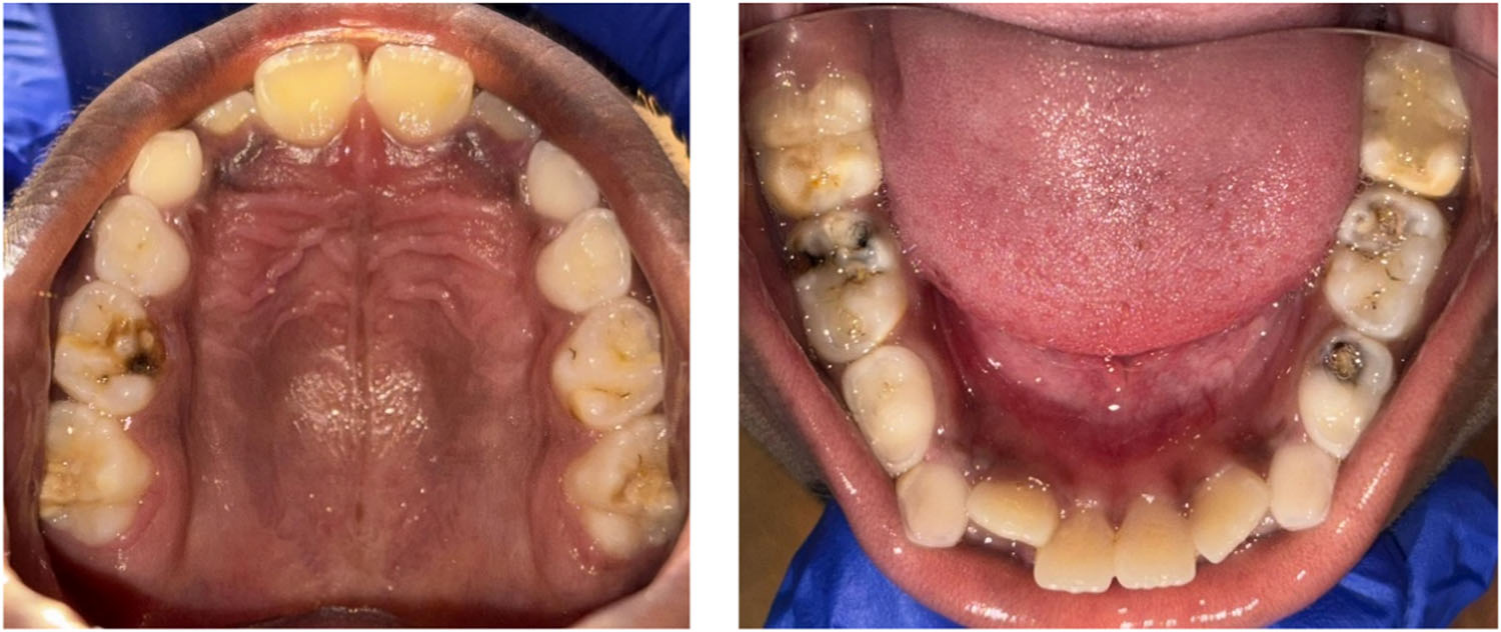
Clinical presentation of hypomineralization in the primary second molar and the permanent first molar of the upper and lower arch of the same individual, respectively.

This exploratory in vitro study involved extracted teeth, encompassing teeth that were both diagnosed with and without hypomineralization in primary second molars. The study's participants were from the Chennai district in Tamil Nadu, India. The participants were children between the ages of 4 and 8 years who did not have any systemic diseases such as type I diabetes or any other syndromes, fever, cold, and other prodromal symptoms. The children were diagnosed using the mDDE/EAPD Index **–** a Tool to diagnose HSPM (Ghanim et al. 2015), following the European Academy of Pediatric Dentistry criteria. Teeth with opacities and posteruptive breakdown with deep caries were included in the study (Figure 1). Teeth with atypical restoration and caries that had destroyed the enamel were excluded.

Other exclusion criteria for this study encompassed children who expressed a preference for pulpectomy procedures, patients who did not have any involvement of deciduous molars, and individuals with carious lesions unrelated to HSPM. This careful selection process ensured that the study cohort included children who fulfilled criteria that closely aligned with the focus and objectives of the research.

### 2.2 Sample Collection and Ethics

Ethical approval for this study was obtained from the Institutional Ethical Committee (IEC‐I/60/2022). The study was explained to the participants and their parents to obtain informed consent. The research process involved data collection, which encompassed a detailed case history of the patients. The case history included information about patient demographics, socioeconomic status (Gunjan Kumar et al. 2022), chief complaints, dental history, medical history, dietary habits, oral hygiene practices, and the pregnancy history of the mother, both pre‐ and post‐partum. Intra‐ and extra‐oral examination was conducted on all participants, whether they had hypomineralization or not. Investigators S.J. and S.S. received training to diagnose HSPM using the EAPD index, to reduce the risk of misdiagnosis and related errors (к = 0.6).

Based on the study carried out by (Mukhtar et al. 2022), sample size was calculated as 10 in each case and control group. Samples of second primary molars, both with and without hypomineralization lesions, were collected for analysis. To preserve the integrity of these tooth samples, they were carefully stored in distilled water containing thymol crystals and preserved at a temperature of −20°C. This storage method ensured the stability of the samples for subsequent analyses and investigations.

### 2.3 Protein Extraction From Teeth

In adherence to the specified inclusion and exclusion criteria, the selected tooth samples were subjected to a precise extraction process. Using a high‐speed handpiece, these teeth samples were sectioned longitudinally into two; using a stereomicroscope, sectioning of enamel pieces from the tooth structure was performed. These enamel pieces were subsequently crushed into a coarse powder using liquid nitrogen and then carefully preserved in Eppendorf tubes for further analysis.

The protein extraction procedure used in this study was based on the methodology recommended by (Porto et al. 2006). To ensure consistency and accuracy, a standardized approach was adopted, involving the use of approximately 20% trichloroacetic acid (TCA) for enamel dissolution. This method allowed for the effective extraction of proteins from the enamel samples, paving the way for subsequent analyses and investigations.

### 2.4 Protein Quantification

**Figure 2:**
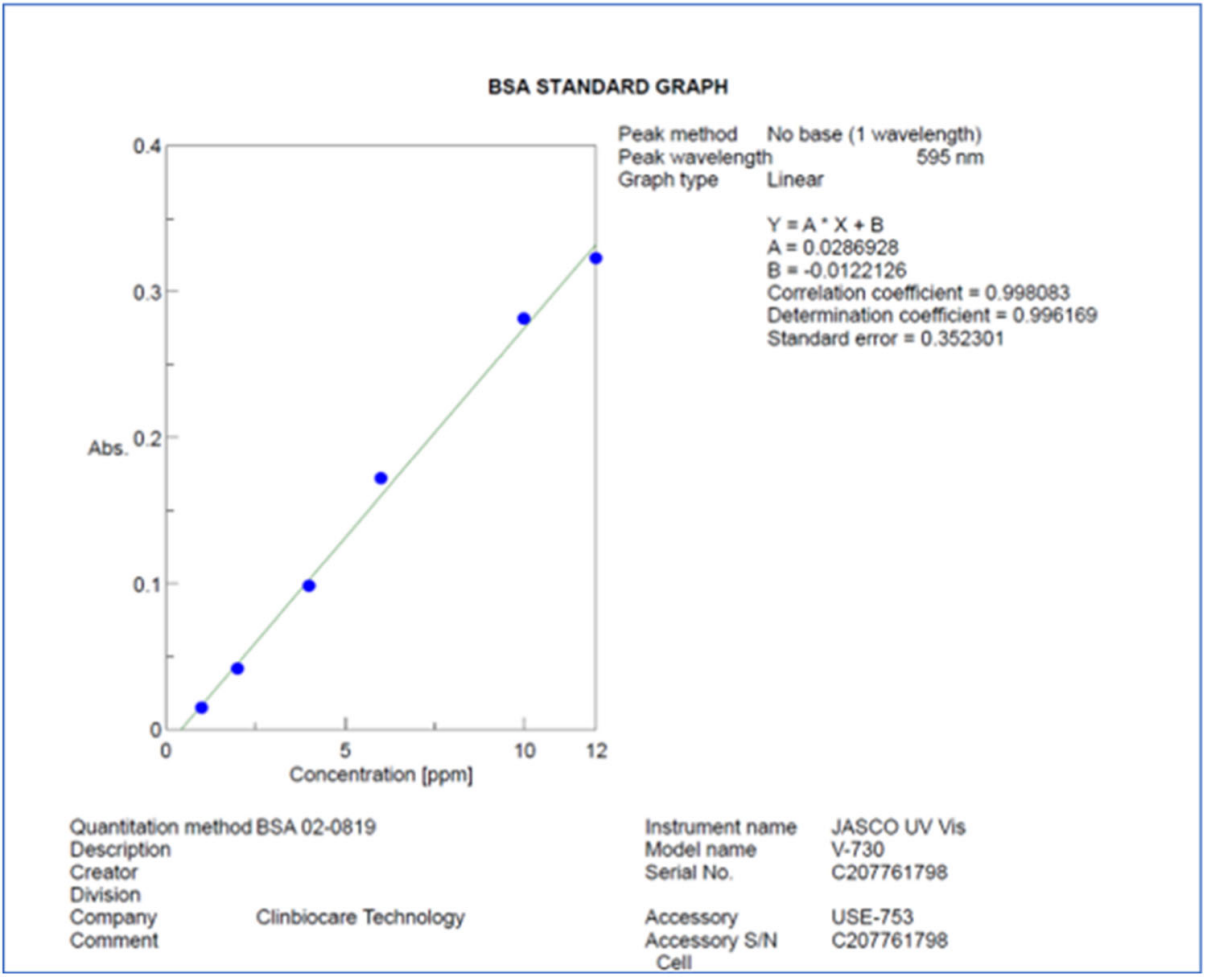
Standard graph for protein quantification.

Total protein quantification was achieved using the Bradford Protein Assay. To determine the protein concentrations in the samples, we constructed a standard graph. In this process, bovine serum albumin (BSA) served as our standard protein reference. The standard graph (Figure 2) was essential for accurate determination of the protein concentrations in our samples.

To create the standards, we prepared known concentrations from a working stock solution of BSA, which had a concentration of 1 mg/mL. We generated a total of seven standards, ranging in concentration from 1 to 12 µg. After preparing the standards, we added the corresponding volumes of each standard to separate tubes and adjusted them to a final volume of 1000 µL using Milli‐Q water. Subsequently, we introduced 1000 µL of Bradford reagent (Sigma‐Aldrich, Germany) to each standard tube. These tubes were then incubated in the dark at room temperature for 10 min. Following the incubation period, we analyzed the samples using a Jasco UV‐Dual Beam Visible Spectrophotometer Model V‐730, Maryland, USA. We utilized the Spectra Manager II analytical program to capture the readings and generate the standard graph.

For the teeth, specimens were processed according to the protocol established by Porto et al. and the supernatants were stored. To calculate the protein content in both diseased and normal teeth samples, we combined 5 µL of the samples with 995 µL of Milli‐Q water. Afterward, we added 1000 µL of Bradford reagent (Sigma‐Aldrich, St. Louis, USA) to each sample. These samples were then incubated for 10 min in the dark at room temperature. Subsequently, we measured the absorbance of these samples at 595 nm using a UV–visible Spectrophotometer (JASCO V‐730, Tokyo, Japan), with BSA serving as the protein standard (Porto et al. 2006).

### 2.5 Gel Electrophoresis and Mass Spectrometry

Sodium dodecyl sulfate‐polyacrylamide gel electrophoresis (SDS‐PAGE) was performed using a 12% polyacrylamide gel mixture, following a procedure adapted from Laemmli, with subsequent staining using Coomassie Blue.

For the preparation of the separating gel, a mixture was created by combining MilliQ water (3.3 mL), 30% acrylamide (4 mL), 15 M Tris pH 8.8 (2.5 mL), 10% SDS (0.1 mL), 10% APS (0.1 mL), and TEMED (0.004 mL) to prepare a total volume of 10 mL. Similarly, the stacking gel was prepared using MilliQ water (2.7 mL), 30% acrylamide (0.67 mL), 1 M Tris pH 6.8 (0.5 mL), 10% SDS (0.04 mL), 10% APS (0.04 mL), and TEMED (0.004 mL) to prepare a total volume of 4 mL.

For the two‐dimensional gel electrophoresis of processed proteins (100 µg), 13 cm IPG strips with a pH range of 3–10 (GE Healthcare, Uppsala, Sweden) were utilized in the first dimension. Protein focusing was carried out under specific conditions, including a total of 44,000 Vhs at a constant temperature of 20°C, following a passive IPG rehydration. The IEF conditions involved a step‐n‐hold at 100 V for 1 h, 300 V step‐n‐hold for 2 h, a gradient to 1000 V for 1 h, a gradient to 5000 V for 3 h, and a step‐n‐hold at 5000 V for 6 h.

Following the first‐dimension IEF, each IPG strip was subjected to an equilibration process. Initially, the strips were placed in a buffer containing 2% DTT, followed by incubation in another buffer, where the DTT was replaced with 2.5% iodoacetamide.

The second‐dimension PAGE (12.5%) was conducted using an SE600 system (GE Healthcare, Uppsala, Sweden) at 1 W/gel for 1 h and 13 W/gel for 3 h. The molecular weight of the relative proteins (10 µg solubilized protein samples) was determined by comparing them to a control protein ladder from Fermentas Inc., MD, USA.

To further analyze the proteins, the Coomassie brilliant blue‐stained gel bands were excised and subjected to digestion with trypsin (GE Healthcare, Uppsala, Sweden). The protein digestion process occurred overnight at 37°C using a trypsin‐to‐protein ratio of 1:50. The reaction was stopped using trifluoroacetic acid. Subsequently, the generated raw data were subjected to further analysis (Izzo et al. 2006).

### 2.6 Data Analysis

Melanie version 9.2.5 was used to analyze the SDS‐PAGE picture, allowing for the extraction of peptides. These peptides were then subjected to mass spectrometry analysis using a SHIMADZU MALDI 7090 instrument that had TOF/TOF MS capability. In order to prepare the samples, the lyophilized material was rehydrated using an 8‐µL solution that had a 50:50 acetonitrile and water mixture. To boost solubility, 0.1% formic acid was added.

About 0.7 µL of this peptide solution was carefully spotted onto a stainless‐steel target plate following vigorous vortexing to ensure homogeneity. A matrix solution was applied above this sample location. A 0.1% trifluoroacetic acid (TFA) solution was added to a 50:50 mixture of acetonitrile and water to create the matrix. After that, the sample site was covered with this matrix solution and allowed to air dry. Mass spectrometry analysis was then carried out in reflectron mode, which is well known for its ability to yield extremely accurate and precise peptide mass determinations. In the mass analysis range, 1–5000 Da, a broad range of peptides and proteins in the sample could be detected.

## 3 Results

Students in the age group of 4–8 years were examined in various parts of Chennai, Tamil Nadu. Of these, 11 HSPM patients whose teeth were scheduled to be exfoliated were selected. Of the 11, 8 were females and 3 were males, and 10 also had MIH alongside HSPM. A total of 27 molars were affected by hypomineralization in 11 individuals. The data of the first 10 HSPM individuals were considered for further analysis.

### 3.1 Demographic Data

In the HSPM group (Group B), it was found that there was an increase in incidence among female patients in comparison to males; 7 were females and 3 were males. The control cohort included 6 females and 4 males. We restricted the present study group to participants in the age span of 4–8 years. A detailed history of the diseased population was collected. The participants in the case‐cohort were mostly from lower middle‐class families (Schwendicke et al. 2018). They mostly complained of pain/sensitivity. No strong correlation between medical history or pre‐ and post‐natal factors was evident. Demographic factors and history of the control cohort and demographic factors and history of the HSPM cohort are provided in Data S1 and S2, respectively.

### 3.2 Intra‐Oral Examination

On the basis of the clinical features of HSPM‐affected individuals, it was evident that most of them had sensitivity in relation to the index teeth molars. A strong association between carious teeth and posteruptive breakdown was evident. Data S3 present the intraoral findings of the HSPM‐affected cohort.

### 3.3 Protein Identification

The results from the Bradford Protein Assay unveiled a five‐fold increase in the protein content in HSPM when compared to their healthy counterparts, as documented in Data S4.

Following the image analysis performed using Melanie 9.2.5, the protein spots were systematically identified and subjected to comprehensive analysis by mass spectrometry. In this detailed scrutiny of the protein profiles, we discovered conspicuous protein spots within the molecular weight range of 30–80 kDa. This specific range notably encompasses a plethora of proteins (*p* < 0.05), including Dentin sialophosphoprotein (DSPP), keratin, type I, Serum Albumin, Anti‐thrombin III, Alpha‐1‐Antitrypsin, Histone H3.2, Actin, Heat shock Protein, Vimentin, Desmoglein‐3, Glyceraldehyde‐3‐phosphate dehydrogenase, Inosine‐5'‐monophosphate dehydrogenase 2, Zinc Alpha 2 glycoprotein, Lysozyme C, Prothrombin, Vit‐D binding Protein, Apolipoprotein A‐1, Defensin 1, Immunoglobulin Gamma, Immunoglobulin Kappa, and Alpha‐Amylase. We observed a notable downregulation of protein‐degrading proteases in HSPM teeth compared to normal teeth (*p* < 0.05). Proteases, including matrix metalloproteinase 20 (MMP20), Histone H1.3, Collagen alpha‐1, and kallikrein 4, showed pronounced downregulation in comparison to the normal teeth.

**Figure 3:**
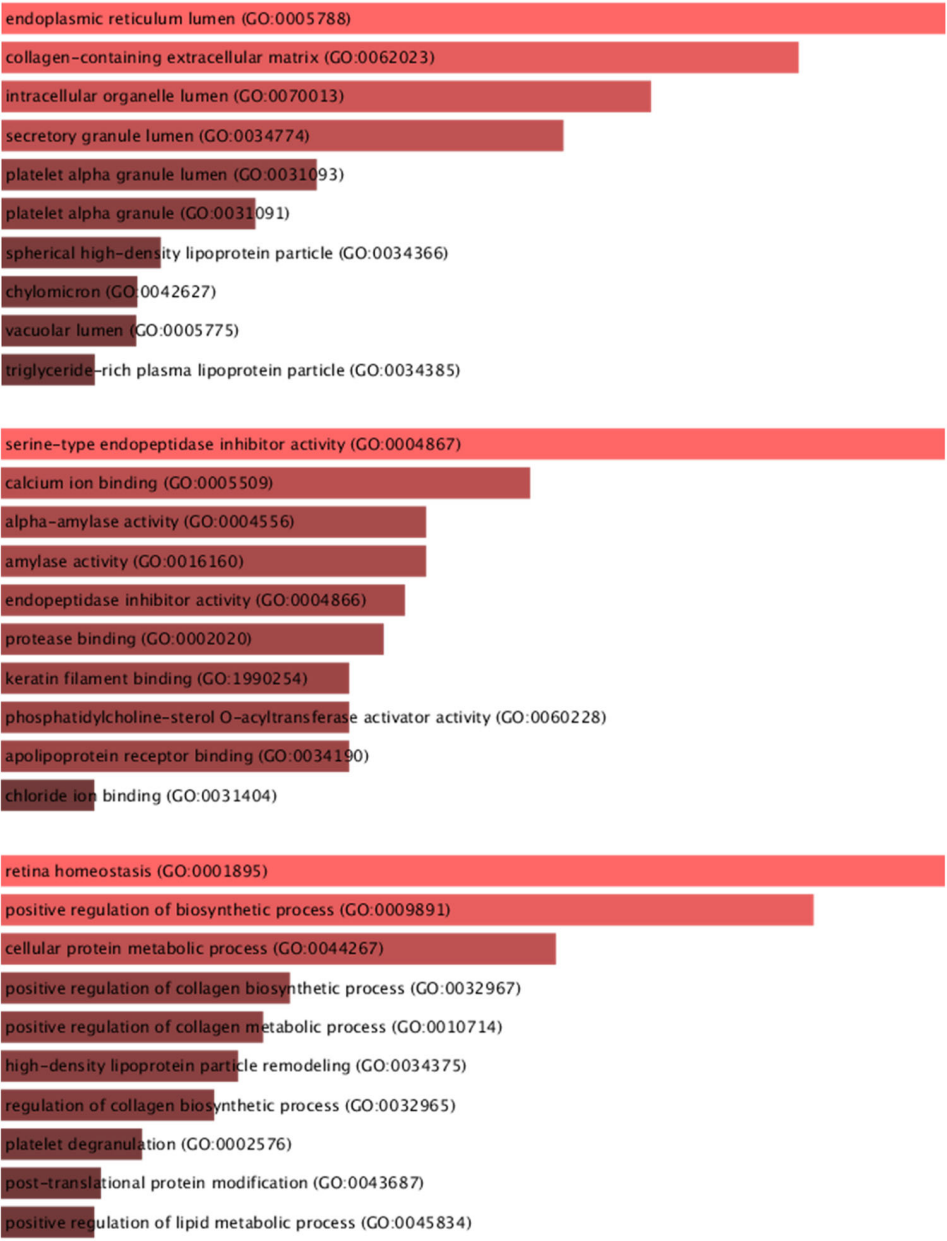
Upregulated gene: cellular components, molecular functions, and biological process.

**Figure 4:**
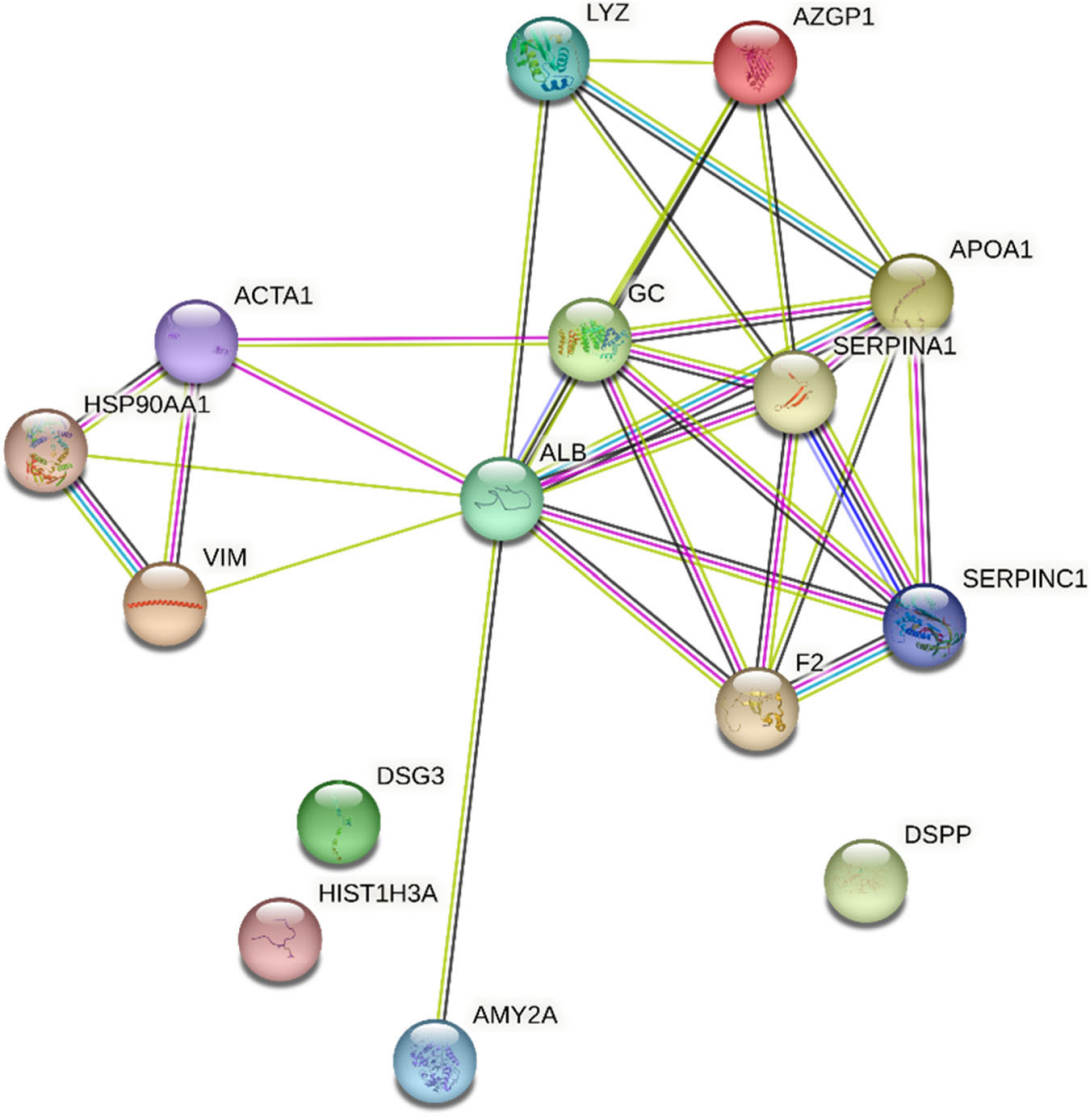
Protein–protein interaction using a string for upregulated proteins in HSPM.

**Figure 5:**
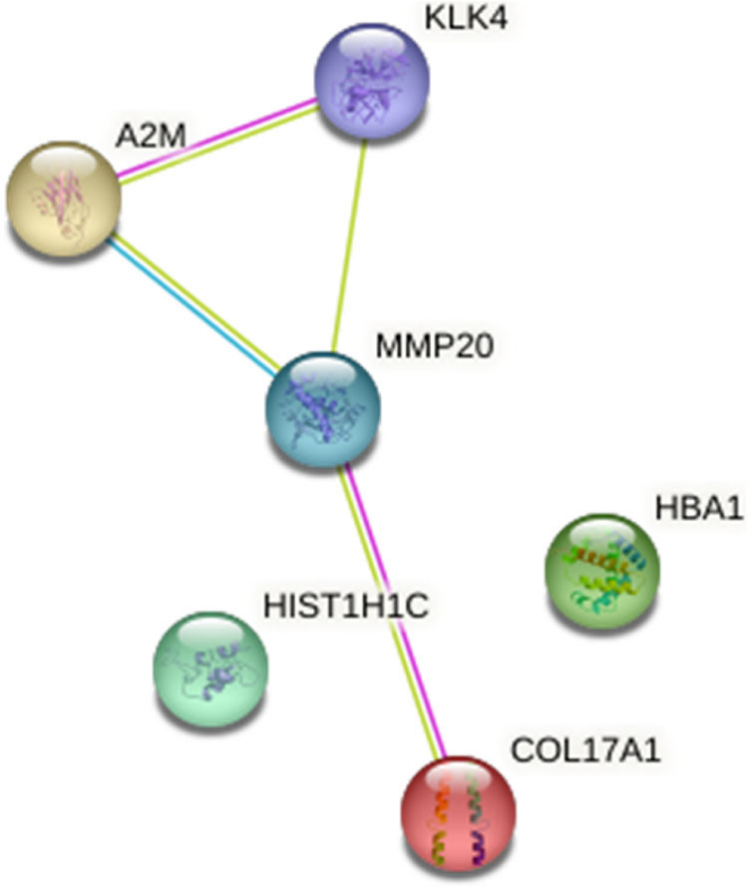
Protein–protein interaction for downregulated proteins in HSPM.

Beyond these specific findings, when viewed in a broader context, the enamel of teeth affected by HSPM consistently demonstrated heightened protein expression levels in contrast to the enamel of healthy teeth. This pattern is clearly shown in Table 1, and the role of these proteins, at the molecular level and in various biological processes alongside their protein–protein interaction, is visually depicted in Figures 3, 4, 5.

**Table 1:** Proteins present in diseased teeth in comparison to normal teeth by MALDI TOF (ANOVA *p*< 0.05).

Proteins	Functions	Mol. wt. (kDa)	% of sequence coverage	Status in HSPM vs. normal teeth
Dentin Sialo‐Phospho Protein	It plays a role in the mineralization process, contributing to the structural integrity of the enamel.	39	26%	Upregulated
Keratin, type 1 (Cytoskeleton)	Provides mechanical support to ameloblasts.	50.818	30%	Upregulated
Serum Albumin	Regulates extracellular osmotic pressure.	67	30%	Upregulated
Antithrombin III	May contribute to the regulation of proteolytic processes in enamel matrix maturation.	58	28%	Upregulated
Alpha‐1‐Antitrypsin	It may protect the enamel matrix from excessive proteolytic degradation during maturation.	52	19%	Upregulated
Histone H3.2	Indirectly influences the expression of enamel‐related genes.	15	32%	Upregulated
Actin	Maintains the cytoskeletal structure in ameloblasts.	42	38%	Upregulated
Heat shock Protein	Protects ameloblasts under stress conditions.	60	42%	Upregulated
Vimentin	A cytoskeletal protein that helps maintain ameloblast structural integrity during matrix secretion and remodeling phases.	57	31%	Upregulated
Desmoglein‐3	Aids in ameloblast adhesion and alignment, essential for uniform enamel layer deposition.	130	34%	Upregulated
Glyceraldehyde‐3‐phosphate dehydrogenase	Primarily a glycolytic enzyme, providing ATP for energy‐intensive processes like enamel matrix secretion and mineralization.	37	31%	Upregulated
Inosine‐5'‐monophosphate dehydrogenase 2	Its activity may support the high cellular activity of ameloblasts during amelogenesis.	55	34%	Upregulated
Zinc Alpha 2 glycoprotein	Modulate energy balance and cellular functions of ameloblasts.	41	31%	Upregulated
Lysozyme C	A bacteriolytic enzyme.	15	27%	Upregulated
Prothrombin	Regulates protease activity during enamel maturation indirectly.	72	26%	Upregulated
Vit‐D binding Protein	Its role in amelogenesis is related to maintenance of calcium and phosphate homeostasis critical for enamel mineralization.	52	20%	Upregulated
Apolipoprotein A‐1	A component of HDL, it may indirectly influence amelogenesis by modulating lipid transport and metabolism essential for cellular processes in ameloblasts.	30	28%	Upregulated
Defensin 1	An antimicrobial peptide that might protect ameloblasts and the enamel matrix from microbial invasion during development.	3.9	40%	Upregulated
Immunoglobulin Gamma	Provides immune defense.	53	23%	Upregulated
Immunoglobulin Kappa	Provides immune defense.	22.5	28%	Upregulated
Alpha‐Amylase	An enzyme that breaks down starch.	56	30%	Upregulated
Matrix Metalloproteinase 20	MMP‐20 cleaves amelogenin and other enamel matrix proteins during the secretory phase.	43	33%	Upregulated
Kallikrein‐related peptidase 4	KLK4 degrades residual enamel matrix proteins, allowing complete mineralization and hardening of the enamel.	33	37%	Upregulated
Histone H1.3	A linker histone that organizes chromatin and regulates gene expression.	32	23%	Upregulated
Alpha‐2‐macroglobulin	A broad‐spectrum protease inhibitor that may regulate the activity of enzymes like MMP‐20 and KLK4 during amelogenesis	725	36%	Upregulated
Hemoglobin subunit alpha	Primarily involved in oxygen transport	15	42%	Upregulated
Collagen alpha‐1 (XVII) chain	This protein helps maintain the integrity of the ameloblast layer, ensuring proper enamel matrix deposition and maturation.	23	32%	Upregulated

*Note:* Proteins hyperexpressed in normal teeth in comparison to HSPM (ANOVA *p*< 0.05).

A few proteins such as Copine‐7, GTPase IMAP family member 4, Chloride intracellular channel protein 2, Syntaxin‐19, Krueppel‐like factor 16, Hepatoma‐derived growth factor‐related protein 2, Zinc finger BED domain‐containing protein 2, Serine/threonine‐protein phosphatase PP1‐beta catalytic subunit, and Recoverin were all novel proteins identified in this study. The expression of these proteins were statistically insignificant (Table 2).

**Table 2:** Other expressed proteins in HSPM with no significant *p* value (*p *< 0.683).

Gene ID	Protein Name	Mol. Wt. (kDa)
CPNE7	Copine‐7	63.21
GIMAP4	GTPase IMAP family member 4	37.738
CLIC2	Chloride intracellular channel protein 2	28.624
STX19	Syntaxin‐19	34.759
KLF16	Krueppel‐like factor 16	25.871
HDGFL2	Hepatoma‐derived growth factor‐related protein 2	74.443
ZBED2	Zinc finger BED domain‐containing protein 2	25.278
PPP1CB	Serine/threonine‐protein phosphatase PP1‐beta catalytic subunit	37.961
RCVRN	Recoverin	23.23

## 4 Discussion

HSPM has the potential to serve as an early indicator of MIH (Quintero et al. 2022). Gaining a better understanding of HSPM's pathophysiology will provide valuable insights into MIH. Given that HSPM involves altered enamel mineralization and/or reduced enamel proteolysis, our study aimed to investigate the distribution of proteins in HSPM in comparison to healthy teeth.

In our research, we observed a slight female predominance in the incidence of this condition. Many patients reported sensitivity of affected teeth, followed by pain during mastication. We also noted a strong association between caries and posteruptive enamel breakdown in HSPM‐affected teeth. Furthermore, our protein analysis using mass spectrometry revealed increased protein expression in HSPM‐affected teeth when compared to healthy teeth (Tables 1 and 2).

Our findings are in agreement with the results of the study carried out by (Mahoney et al. 2004) that focused on the elasticity and hardness of HSPM‐affected teeth in comparison to normal teeth. Similar to their conclusions, our research indicated that hypomineralized teeth showed weaker enamel, evident from increased protein content compared to normal teeth. This suggests compromised mineralization, making HSPM‐affected teeth more susceptible to structural deterioration.

The observed protein alterations in HSPM‐affected teeth provide valuable insights into the condition's pathogenesis, which can inform future diagnostic and therapeutic strategies in dental care. In our study, we also noted a substantial five‐fold increase in the total protein content in HSPM‐affected teeth compared to normal teeth. A similar investigation by Farah et al. (2010) reported an even more significant increase, with 8–21 times higher protein levels in enamels affected by MIH in contrast to normal enamels. It is important to note that due to the smaller size of primary molars in comparison to permanent molars, the absolute amount of extracted protein was relatively lower quantitatively. This increase in protein content parallels with what is observed in other enamel defects related to maturation and calcification, where the organic matter outweighs the mineral components.

One noteworthy protein in our findings is Dentin Sialophosphoprotein (DSPP), which is commonly found in odontoblasts and presecretory ameloblasts. In situ hybridization studies have indicated that DSPP plays a significant role in substantial matrix production and dentinogenesis. The expression of DSPP also suggests active ameloblast activity. Additionally, our results demonstrated the presence of copine‐7 (CPNE7), a protein previously observed in a study carried out by (Park et al. 2018). This protein is believed to play a crucial role in enamel matrix formation, further underscoring its relevance in HSPM. Furthermore, our study identified the upregulation of Antithrombin III and alpha‐1‐antitrypsin, both of which belong to the serpin family of proteins. These proteins are elevated in HSPM and are known to inhibit the activity of KLK4, resulting in decreased proteolytic activity and reduced biosynthesis of these proteases. The combined effect of Antithrombin III and alpha‐1‐antitrypsin leads to the inhibition of KLK4 activity (Gil‐Bona and Bidlack 2020).

Furthermore, the upregulation of Antithrombin III and alpha‐1‐antitrypsin underscores their involvement in regulating proteolytic activity within the context of HSPM. These findings deepen our understanding of the molecular mechanisms at work in HSPM and related dental enamel conditions. Histone 3.2, a component of nucleosomes primarily involved in transcription, DNA replication, repair, and chromosomal stability, was identified in our study. Its role is closely associated with mitosis during spindle attachment to the centromere (Henikoff and Smith 2015). Additionally, other proteins such as Desmoglein‐3, GAPDH, and Inosine‐5'‐monophosphate dehydrogenase are known to be influenced by stress (Rehman et al. 2019). However, the specific role of stress and the circadian rhythm in modulating protein expression in HSPM has not been extensively studied. The observed protein expression patterns, often associated with stress, suggest a potential link between stress, the circadian rhythm, and the physiological function of proteins involved in tooth formation (Papagerakis et al. 2014).

We also observed downregulation of proteases such as MMP20 and KLK4, which are responsible for the extracellular processing of Amelogenins and non‐amelogenins. These enzymes are typically present in higher quantities in healthy enamel, but are downregulated in HSPM. This suggests impaired proteolytic activity in individuals with HSPM, consistent with findings from studies carried out by (Farah et al. 2010 and Mukhtar et al. 2022). Identification of Serum Albumin further substantiates the pathophysiology proposed by (Hubbard et al. 2021), indicating the role of serum albumin replacing amelogenin, further increasing the potential decrease of function of proteases causing mineralization poisoning. Technical challenges related to the low hard tissue content in deciduous molars, compared to permanent molars, may have limited the identification of various other proteins in the teeth samples. It is worth noting that the presence of keratins, filamentous cell proteins, may be attributed to contamination.

Our findings collectively indicate that HSPM‐affected teeth have increased protein content and reduced mineral content compared to healthy teeth. Proteolytic activity is hindered due to decreased protease activity and increased protease inhibitor activity. Detailed studies on these protein‐coding genes, their expression patterns, and functional activities could provide a more precise understanding of the underlying causes of hypomineralization, particularly in molars and incisors. However, in this study, there is a limitation of possible contamination from the salivary proteins. In addition, absolute quantification of the proteins was not achieved through this process.

On the basis of our findings, it appears that Hypomineralized second primary molars (HSPMs) could potentially serve as an early warning sign for MIH. A substantial increase in the protein content within HSPM points toward a possible genetic influence. This research not only provides avenues for future genomic investigations but also underscores the critical importance of early intervention in preserving permanent dentition from enamel deterioration. Additionally, it highlights the significance of studying the genetic factors underlying these dental conditions for improved diagnosis and prevention.

## Author Contributions

Project ideology and methodology: Ramya Sekar and Snehashish Ghosh. Principal investigator and project administration: Sharon Jessica. Manuscript assessment: Kavitha B. and Safal Dhungel. Project implementation: Mahesh Ramakrishnan. Tables and figures: Shazia Fathima. Statistics and Wet laboratory procedures: Monisha Prasad. Sampling: Jaiganesh I. Sample collection: Sindhu Subramani.

## Ethical Statement

Meenakshi Ammal Dental College and Hospital's Ethical Committee approved the study, which was carried out in compliance with the Declaration of Helsinki (MADC/IEC‐I/60/2022‐1). All dental samples were obtained with Institutional Ethical Committee approval. All study participants provided written informed consent after being informed about the study by the patient's attendant.

## Conflicts of Interest

The authors declare no conflicts of interest.

## Supporting information

Supporting information.

## Data Availability

The data sets used and analyzed in the present study are available from the corresponding author upon reasonable request.
