# Supplementary material for: Differential Expression of Hard Tissue Proteins in Hypomineralized Second Primary Molars in Comparison to Normal Teeth
Source: Clin Exp Dent Res. 2025 Feb 3;11(1):e70079. doi: 10.1002/cre2.70079 (PMC11789269; doi:10.1002/cre2.70079)
Supplement: Supplementary file 1 — Supporting information. [file CRE2-11-e70079-s001.docx]

**1:Demographic factors and History among control cohort**

| **Age** | **Gender** | **Socio– economic status [37]** | **Chief complaint** | **Medical History** | **Dental History** | **Pre/post natal factors** | **Family History** |
| --- | --- | --- | --- | --- | --- | --- | --- |
| 8 | M | Upper middle | Check-up | No relevant history | - | Infection | Absent |
| 6 | F | Upper middle | Check-up | Asthma | - | Absent | Absent |
| 6 | M | Lower middle | Check-up | No relevant history | - | Fever | Present |
| 5 | F | Upper | Check-up | No relevant history | - | Fungal infection | Absent |
| 8 | F | Upper middle | Check-up | No relevant history | - | Absent | Absent |
| 8 | F | Upper middle | Check-up | No relevant history | - | Absent | Absent |
| 7 | F | Upper middle | Check-up | No relevant history | - | infection | Present |
| 8 | M | Upper | Check-up | No relevant history | - | Absent | Absent |
| 5 | M | Upper middle | Check-up | No relevant history | - | Cough | Absent |
| 8 | F | Lower middle | Check-up | No relevant history | - | Absent | Absent |

**2:Demographic factors and History among HSPM cohort**

| **Age** | **Gender** | **Socio– economic status[-]** | **Chief complaint** | **Medical History** | **Dental History** | **Pre and post natal factors** | **Family History** |
| --- | --- | --- | --- | --- | --- | --- | --- |
| 7 | F | Lower middle | Sensitivity | No relevant history | Extraction | Fever with antibiotics | Present |
| 8 | F | Upper middle | Sensitivity | Asthma | - | Diarrhoea | Absent |
| 6 | F | Lower middle | Pain | No relevant history | Restoration | Fever with antibiotics | Absent |
| 8 | M | Upper lower | Sensitivity | No relevant history | Restoration | Fungal infection underwent treatment | Absent |
| 6 | F | Upper lower | Pain & Sensitivity | No relevant history | - | Fungal infection | Present |
| 8 | M | Upper middle | Pain | No relevant history | Extraction | Fever | Absent |
| 8 | F | Upper lower | Sensitivity | Asthma | Restoration | Fever with antibiotics | Present |
| 7 | F | Upper lower | Pain | No relevant history | - | NIL | Present |
| 5 | M | Lower | Sensitivity | No relevant history | - | Antibiotic usage (kid) | Present |
| 8 | F | Lower | Pain & Sensitivity | No relevant history | Restoration | NIL | Present |

**3: Intraoral findings of the HSPM affected cohort**

| **S.No** | **Sensitivity** | **Opacity/ discoloration** | **Post-Eruptive Breakdown** | **Caries** | **Restoration(not associated with extracted teeth)** | **Extraction** |
| --- | --- | --- | --- | --- | --- | --- |
| 1 | Present | Present | Present | Present | - | - |
| 2 | Present | - | Present | - | - | - |
| 3 | - | Present | - | Present | Present | - |
| 4 | Present | Present | Present | - | Present | Present |
| 5 | Present | - | - | Present | - | - |
| 6 | - | Present | Present | Present | - | Present |
| 7 | Present | - | Not evident | Present | Present | - |
| 8 | - | Present | Present | Present | - | - |
| 9 | Present | - | - | Present | - | - |
| 10 | Present | Present | Present | Present | Present | - |

4 : **Protein concentration in normal and diseased enamel quantified through three methods.**

| Groups | Number of samples | Sample ID | Amount of Total Protein | Number of Proteins |
| --- | --- | --- | --- | --- |
| Group A(HSPM) | 10 | S1 | 10.33 | 18 |
| Standardised to yellow to brown lesion |  | S2 | 11.02 | 22 |
|  |  | S3 | 10.05 | 20 |
|  |  | S4 | 9.08 | 16 |
|  |  | S5 | 11.35 | 27 |
|  |  | S6 | 10.3 | 17 |
|  |  | S7 | 11 | 22 |
|  |  | S8 | 9.08 | 16 |
|  |  | S9 | 10.05 | 20 |
|  |  | S10 | 11.0 | 20 |
| Group B (Normal Teeth) | 10 | S11 | 2.23 | 7 |
|  |  | S12 | 1.59 | 5 |
|  |  | S13 | 0.21 | 3 |
|  |  | S14 | 0.87 | 3 |
|  |  | S15 | 1.36 | 5 |
|  |  |  |  |  |
|  |  | S16 | 1.01 | 5 |
|  |  | S17 | 0.84 | 5 |
|  |  | S18 | 1.32 | 7 |
|  |  | S19 | 0.54 | 3 |
|  |  | S20 | 1.21 | 6 |
